# Supplementary material for: Are Cactus Spines Modified Leaves? Morphological and Anatomical Characterization of Saguaro Seedlings (Carnegiea gigantea) with Special Focus on Aerial Organ Primordia
Source: Plants (Basel). 2024 Dec 4;13(23):3406. doi: 10.3390/plants13233406 (PMC11644746; doi:10.3390/plants13233406)
Supplement: Supplementary file 1 [file plants-13-03406-s001.zip › plants-3203525-supplementary.pdf]

SUPPLEMENTARY MATERIAL

Miravel-Gabriel, et al.,

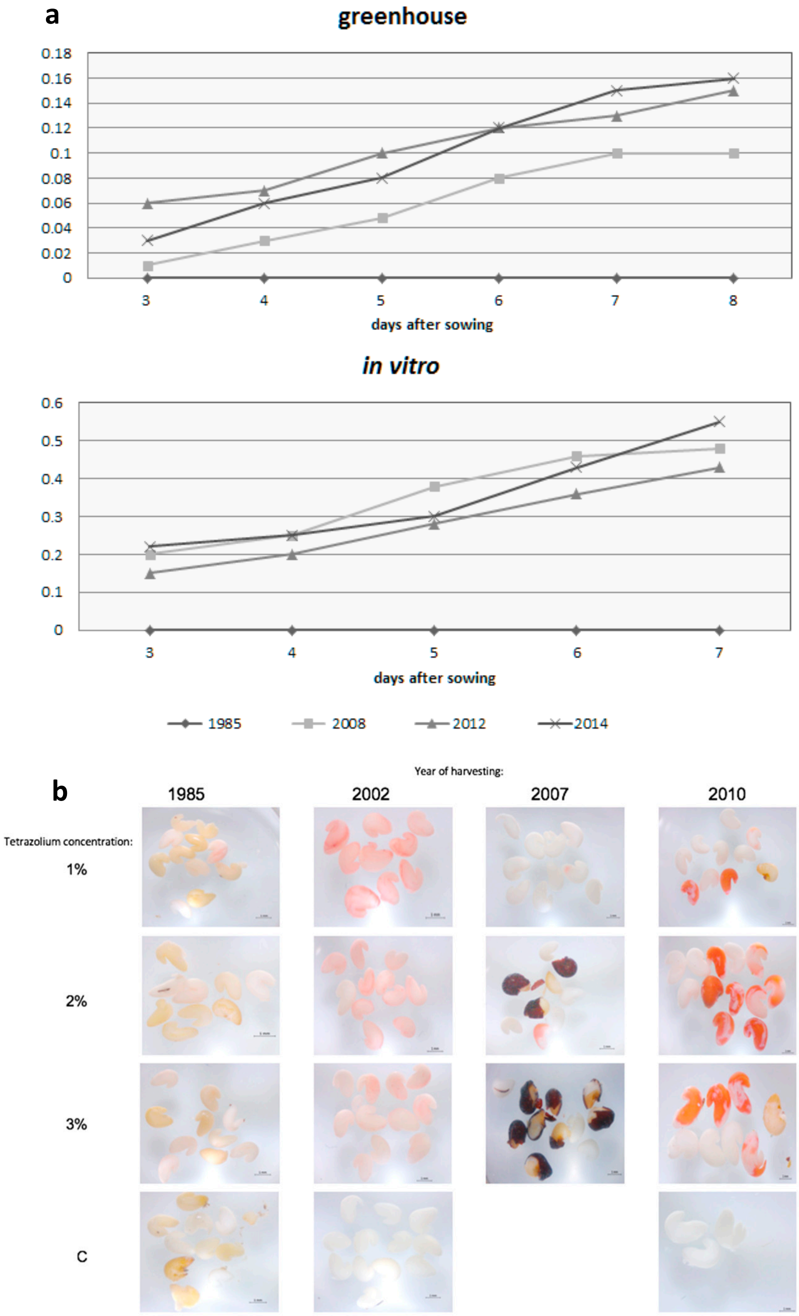

Figure S1. **Germination rates and viability of saguaro seeds.** a) Germination rates under greenhouse (soil) and *in vitro* conditions. Different batches of seeds were tested corresponding to different collection years. b) Viability tests using Triphenyl Tetrazolium chloride (TTC) test. Three different concentrations of TTC and seeds collected in four different years were evaluated. c = control (no treatment).

SUPPLEMENTARY MATERIAL

Miravel-Gabriel, et al.,

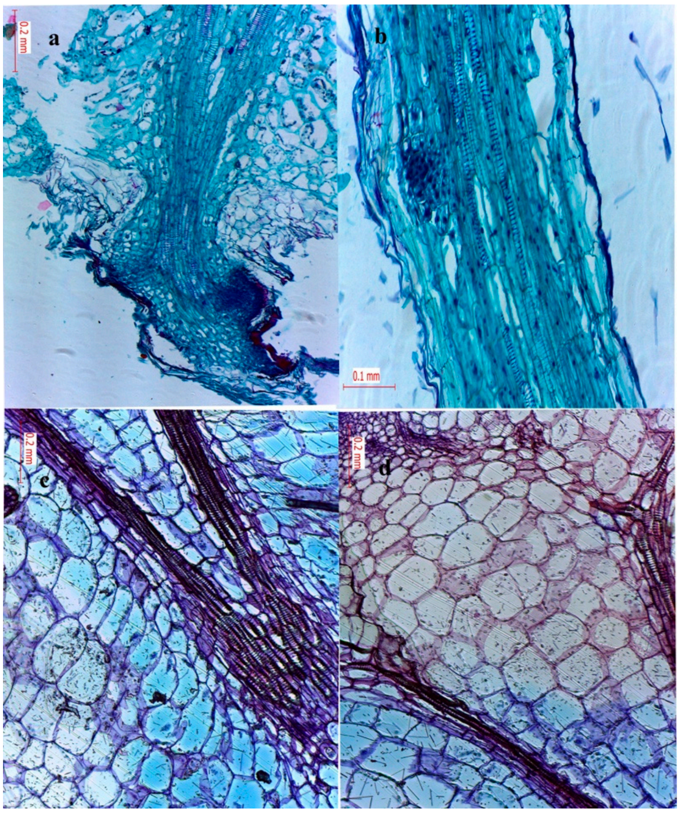

Figure S2. Histological longitudinal sections of saguaro seedlings showing early vasculature. (a) Hypocotyl-root transition, (b) primary root, (c) hypocotyl (middle part), (d) hypocotyl (upper part).

Table S1. Tetrazolium test for seed viability

Results of the Tetrazolium chloride test.
1985

SUPPLEMENTARY MATERIAL

Miravel-Gabriel, et al.,

	1%	2%	3%	Control (-)
Stained:	1	0	2	0
Total:	11	11	11	11
% viability:	9%	0%	18.1%	0%
2002				
Stained:	10	10	6	0
Total:	10	11	11	10
% viability:	100%	90.9%	54.5%	0%
2007				
Stained:	0	1	0	0
Total:	10	8	11	11
% viability:	0%	12.5%	0%	0%
2010				
Stained:	4	8	4	0
Total:	10	12	8	3
% viability:	40%	66.6%	50%	0%
